# The Combined Utility of HBME-1 and Galectin-3 Immunohistochemistry and BRAF V600E Mutations in the Diagnosis of Papillary Thyroid Carcinoma

**DOI:** 10.7759/cureus.20339

**Published:** 2021-12-10

**Authors:** Subramaniam Ramkumar, Shanthakumari Sivanandham

**Affiliations:** 1 Pathology, Woodland Hospital, Shillong, IND; 2 Pathology, PSG Institute of Medical Sciences & Research, Coimbatore, IND

**Keywords:** follicular variant of papillary thyroid carcinoma, follicular adenoma, thyroid neoplasms, papillary thyroid carcinoma, brafv600e, galectin-3, hbme-1, malignancy, thyroid

## Abstract

Newer diagnostic modalities have revolutionized the pathologist’s approach to diagnosing thyroid malignancies. Molecular characterization of these malignancies has helped circumvent common morphologic diagnostic difficulties by integrating their genotypic, phenotypic, and immunohistochemical features. *BRAF* V600E mutation has been characterized as highly specific for thyroid carcinoma, especially papillary thyroid carcinoma (PTC); human bone marrow endothelial marker-1 (HBME-1) and galectin-3 are also such markers that are highly specific for PTC. We propose to study HBME-1 and galectin-3 expression and *BRAF* V600E mutation in thyroid neoplasms and do a comparative analysis to determine whether there is a correlation between *BRAF* V600E expression and that of HBME-1 and galectin-3. We further propose to analyze the individual and combined diagnosed utility of the above-mentioned ancillary diagnostic techniques.

## Introduction

Diseases of thyroid are common worldwide. Thyroid disorders are also common in India and are on the rise. From various studies done in the past, it has been calculated that about 42 million Indians suffer from thyroid disorders [1]. The Indian Council of Medical Research established the National Cancer Registry Program (NCRP), which has collected the data of more than 300,000 cancer patients between the years 1984 and 1993. The nationwide relative frequency of thyroid cancer among all the cancer cases was 0.1-0.2%. The age-adjusted incidence rates of carcinoma of the thyroid per 100,000 population are about 1 for males and 1.8 for females according to the Mumbai Cancer Registry. This covered a population of 9.81 million subjects [1].

Nearly 95% of thyroid neoplasms originate from follicular epithelial cells. From a hospital-based cancer registry in India covering 1185 new cases of thyroid neoplasms, it was shown that the commonest epithelial neoplasm arising from follicular cells is papillary carcinoma (85%), followed by follicular neoplasms [2]. 

Papillary thyroid carcinomas (PTC) are characterized by distinct nuclear features and usually have a favorable prognosis. Follicular thyroid carcinomas are less frequent and have a worse prognosis because of hematogenous dissemination [3]. Microscopically, papillary carcinoma is composed of true papillae with thin fibrovascular cores. These papillae are lined by stratified columnar or cuboidal cells with nuclear overlapping, crowding, nuclear grooves, inclusions, and clearing. These nuclear features are diagnostic of papillary carcinomas. These neoplasms spread through lymphatics and nearly a significant half of these cases present with lymph node metastases at the initial presentation. Papillary microcarcinomas are incidental findings when the thyroidectomy is done for other indications. These lesions are defined by the size, which is 1cm or less in diameter. Grossly, these lesions are mostly unencapsulated and occasionally present with metastasis to regional lymph nodes [4]. The presence of follicular structures with nuclear features characteristic of PTC and the presence of capsular invasion is in favor of a follicular variant of PTC. These lesions are prone to vascular invasion and metastasis to distant sites [5]. The above three morphologic entities are commonly encountered controversial entities due to subtle morphologic overlaps with reactive papillary hyperplasia and solitary follicular nodule with papillary-like nuclear features. In such scenarios, the pathologist depends on ancillary techniques like Immunohistochemistry (IHC) and molecular studies for an accurate diagnosis. A recent study in India also stressed the importance of the usage of ancillary studies such as IHC and genetic profiling of thyroid neoplasms in the near future on a routine basis [1]. The tall cell variant, oncocytic variant, Warthin’s tumor-like variant, cribriform variant, diffuse sclerosis variant, clear cell variant are rare variants but rarely controversial due to their distinct morphologic characterizations [5,6,7].

## Materials and methods

All cases diagnosed as thyroid neoplasms in the past decade from the department of pathology were considered for the study. After obtaining permission from the authorities and due clearance from the Institutional Human Ethics Committee, PSG Institute of Medical Sciences and Research, Coimbatore, India, the clinical details of these cases were taken from the medical records department of the institute. Age, sex, clinical presentation, hormone status, and tumor, nodes, and metastases (TNM) status were obtained by analyzing the case records. Haemotoxylin and Eosin (H&E) stained slides were analyzed for the following microscopic features: (i) architectural pattern, (ii) nuclear atypia, (iii) vascular invasion, (iv) capsular invasion/extrathyroidal extension, (v) mitotic activity, (vi) necrosis, (vii) presence of amyloid deposits in case of medullary carcinoma, (viii) staging done according to TNM classification.

Paraffin blocks of those sections that had high tumor density with less normal thyroid tissue were included for the study by using H&E stained slides. Tissue blocks with large areas of hemorrhage, cystic change, and necrosis were excluded from the study. For IHC, a dextran polymer detection system was used. Antigen retrieval was done with pressure cooker using citrate buffer (pH 6.0). Peroxidase and power blocks were done. After this, primary antibodies human bone marrow endothelial marker-1 (HBME-1) and galectin-3 were applied. Then secondary antibody, i.e. polymer horseradish peroxidase, was applied. Colour development with working color development solution was done. The slides were then assessed for staining intensity. From the same tissue blocks used for HBME1 and galectin-3, DNA was extracted and *BRAF* V600E mutational analysis was done.

Details of antibodies used

HBME-1

Source: mouse monoclonal; Tested Reactivity: human, formalin-fixed paraffin-embedded (FFPE); Localization: cytoplasm; Dilution: Ready to use; Supplier: PathnSitu, Livermore, California, United States (US).

Galectin-3

Source: mouse monoclonal; Clone: B2C10; Class: IVD; Isotype: mouse IgG1; Tested Reactivity: human, FFPE; Localization: cytoplasm; Dilution: Ready to use; Supplier: PathnSitu, Livermore, California, US.

Tissue preparation from FFPE blocks

Using a pen, the area of the tissue containing the maximum tumor was marked on H&E stained slide. From the FFPE tissues, 10-15 μm sections were cut and placed over the plain glass slide. The sections were deparaffinized by immersion in xylene, followed by dehydration in graded alcohol. The H&E stained slide with the marked tumor area was kept over the unstained section slide. Using the circled area of interest on the unstained tissue section slide as a guide, a clean scalpel blade was used to scrape the tissue in the area containing tumor tissue. The tissue was placed in a 2ml Eppendorf Tube.

Isolation of DNA

The tissue was incubated at 50^o^C until it gets dried up completely. To the dried tissue, 500 µL lysis buffer and 35 µL Proteinase K (20mg/mL) were added, mixed well, and the solution incubated at 60^o^C for two to three hours until the tissue gets dissolved. The temperature was raised to 95^o^C and the tubes were incubated for eight minutes to inactivate Proteinase K. Contaminating RNA was removed by addition of 15 µL Ribonuclease (RNase) (10mg/mL) and incubated at 37^o^C for 15 minutes. To each tube vortex, 1ml of a phenol-chloroform mix (1:1 ratio) was added and centrifuged at 13,000 RPM for five minutes. The supernatant was transferred to fresh tubes to which an equal volume of chloroform was added, vortexed, and centrifuged at 13,000 RPM for five minutes. The supernatant was transferred to fresh tubes. DNA was precipitated by adding 1mL of 100% ethanol and 50 µL of 30M sodium acetate. Further, the solution was centrifuged at 13,000 RPM for five minutes to pellet down DNA. To the pellet, 1ml of 75% ethanol was added and centrifuged at 13,000 RPM for five minutes. The pellet was dried and suspended with 35 µL of Tris-ethylenediaminetetraacetic acid (TE) buffer.

Polymerase chain reaction (PCR) for amplification of exon 15 of *BRAF* gene

**Figure 1:**
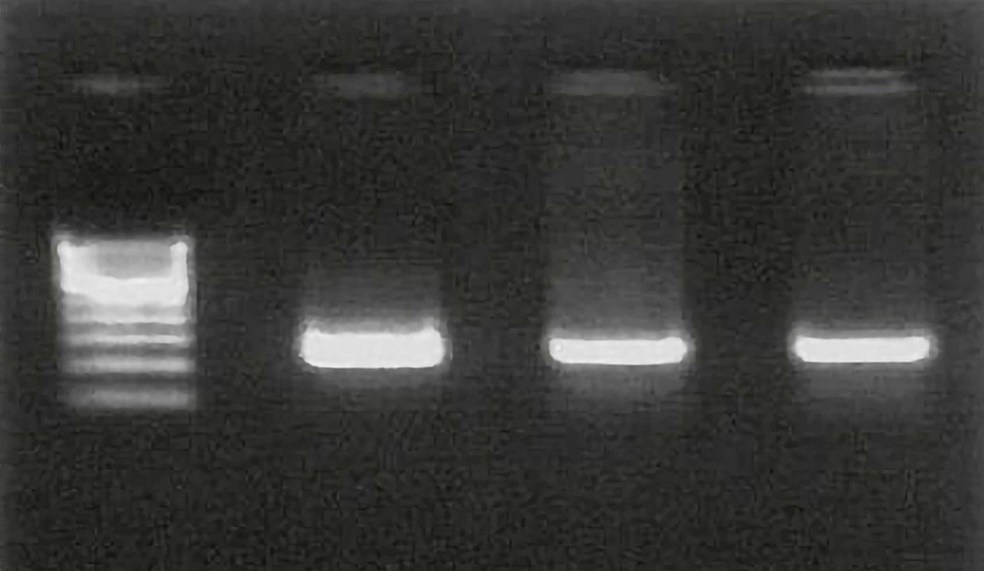
Agarose gel electrophoresis: PCR amplification by using 15 F and 15 R primers. The PCR product size is 224 bp. PCR: polymerase chain reaction

PCR was executed for amplification of 224 bp fragment of the exon 15 of *BRAF* gene using DNA isolated from FFPE tissues as a template and *BRAF* 15F and 15R primers. PCR reactions were performed in 20 µl of 1.5mM MgCl2 with 200µM deoxynucleoside triphosphate, 50-100 ng of DNA, 0.5 µM of each primer, and 2.5 U Taq polymerase. PCR was carried out for 40 cycles with denaturing at 95^o^C, annealing at 59^o^C, and extension at 72^o^C. PCR amplification was confirmed on 2% agarose gel with an expected PCR product size of around 224 bp (Figure 1 ).

Clean-up of PCR product

PCR products were cleaned up using HiPurA™ PCR Product Purification Kit (HiMedia Laboratories Pvt Ltd, Mumbai, India). As per manufacturer instructions, the procedure was followed: (i) PCR product was taken in a 1.5 mL tube; (ii) 4-5 volume of PCR binding solution (SPB) was added; (iii) mixed thoroughly by gentle pipetting, spinning the tube; (iv) lysate was loaded in Miniprep spin column and centrifuged at 10,000 X g for a minute at room temperature. Discarded flow through; (v) washed with 700µL of diluted wash solution (HPE) and centrifuged at 10,000 X g for a minute at room temperature. Discarded flow through; (vi) 500 µL of diluted wash solution (HPE) was added and centrifuged at 10,000 X g for a minute at RT. Discarded flow through; (vii) the empty tube was centrifuged for two minutes at 13000 X g to dry column matrix; (viii) column transferred to new 2 mL tube, added 30 - 50 µL of elution buffer; (ix) centrifuged one minute at 13000 X g to elute DNA; (x) DNA was quantified using NanoDrop.

Restriction fragment length polymorphism (RFLP)

For genotyping, RFLP was carried out using TspRI enzyme (Thermo Fisher Scientific Corp., Waltham, Massachusetts, US). This enzyme cuts the wild-type PCR product to give two bands of size 125 bp and 87 bp, respectively.

**Figure 2:**
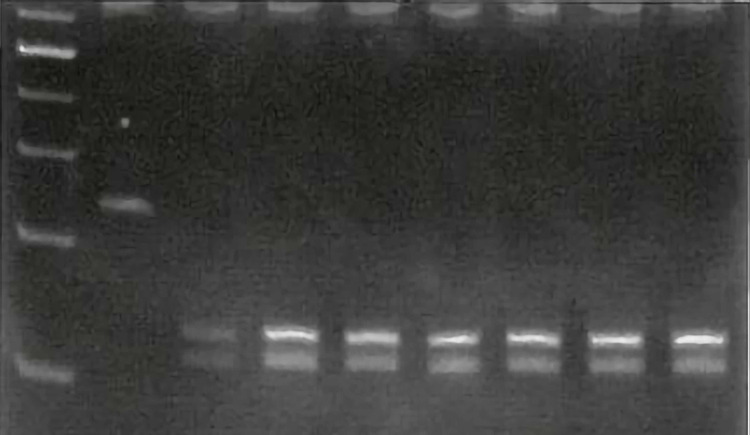
Polyacrylamide gel electrophoresis: Restriction enzyme digestion. Control: Well no. 1: 224 bp. Wild type: Well no. 2, 3, 4, 5, 6, 7, 8 (120bp + 104bp)

Approximately 0.3 µg of PCR product was added to 30 µl reaction mix containing 2 µl of 10x FastDigest green buffer (Thermo Fisher Scientific Corp., Waltham, Massachusetts, US) and 1 µl of FastDigest enzyme. The reaction mix with PCR product was incubated at 65⁰C for 1.5 hours and the product was resolved using 12% polyacrylamide gel electrophoresis (PAGE). PAGE was performed using Amersham ECL Gel Horizontal Electrophoresis System (GE Healthcare, Chicago, Illinois, US) at 100 V for about five hours and the gel was stained with ethidium bromide. The stained gel was viewed under UV and documented using a gel documentation system. The wild-type homozygous alleles showed two bands around 125 bp and 87 bp, whereas mutant alleles showed one band around 212 bp. Heterozygous was distinguished from wild-type and mutant homozygous by the presence of three bands (212 bp, 125 bp, and 87 bp) (Figure 2).

## Results

The Department of Pathology received 119 cases of thyroid neoplasms in the past decade, giving an overall incidence of 2.5%. Out of these, 66 cases were selected using the inclusion and exclusion criteria mentioned earlier. This incidence rate is significantly higher than that reported in Western literature and is similar to the report given by the Regional Cancer Registry in Chennai, India [1]. Papillary thyroid carcinomas constituted 50% (33 out of 66 cases) of all thyroid neoplasms, followed by follicular neoplasms, which constituted around 44% of cases (29 out of 66 cases). Medullary thyroid and anaplastic thyroid carcinomas constituted around 6% of all thyroid carcinomas. In our study, a significant number of cases (46 out of 66 cases) were between the fourth to sixth decades, and 41 cases were Stage I (Table 1), which is in agreement with the literature. As most of the cases were in Stages I and II, HBME-1 and galectin-3 were also found more frequently in Stages I and II lesions. These findings could be explained by the fact that all cases were detected early, and surgery was performed at an early stage.

**Table 1:** Stages of thyroid neoplasms selected in the study.

Stage	Number of cases
Stage 1	41
Stage 2	11
Stage 3	8
Stage 4	6

**Figure 3:**
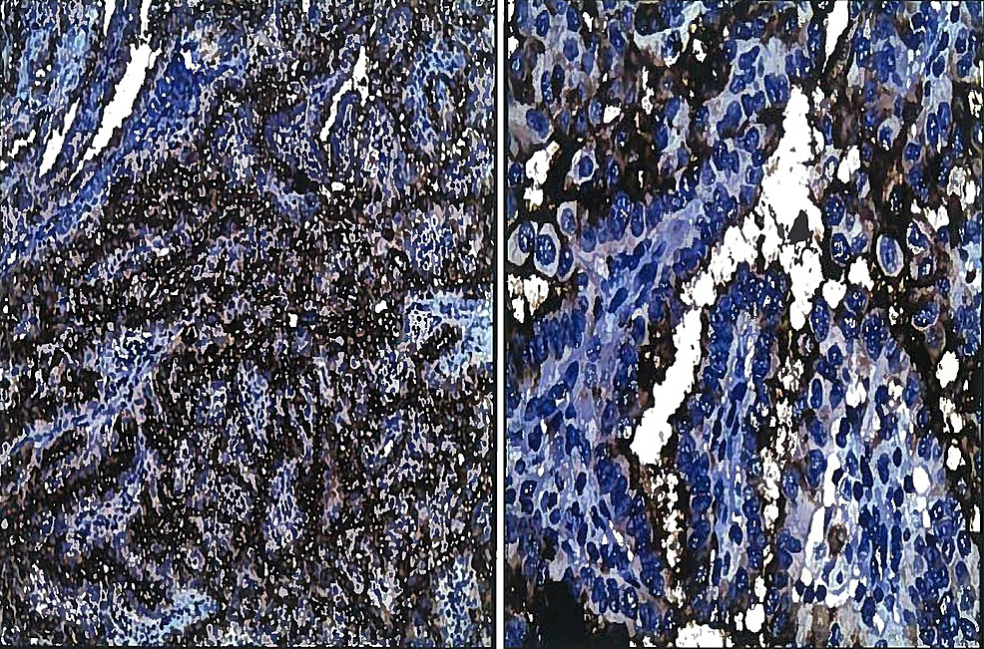
Papillary thyroid carcinoma: the cells show strong immunostaining for HBME-1 (Left: IHC x100, Right: IHC x400). HBME-1: human bone marrow endothelial marker-1; IHC: immunohistochemistry

**Figure 4:**
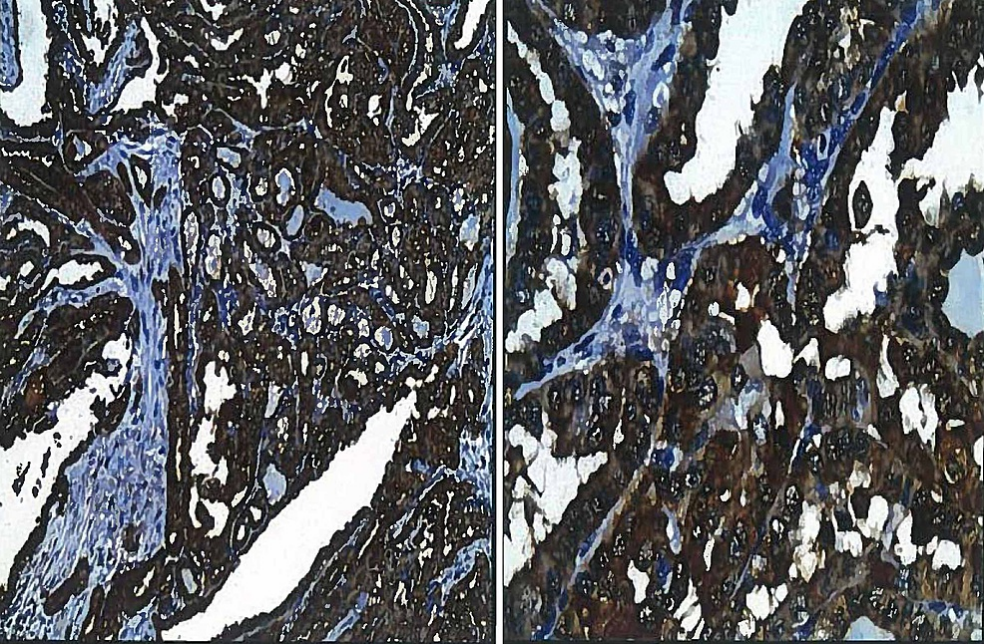
Papillary thyroid carcinoma: the cells show strong immunostaining for galectin-3 (Left: IHC x100, Right: IHC x400). IHC: immunohistochemistry

IHC analysis for HBME-1 and galectin-3 was performed on all 66 cases (Figures 3-4).

The findings were analyzed and correlated with the type of neoplasm (Table 2) and the presence of *BRAF* mutation. The data obtained was further stratified, studied in detail, and analyzed. These analytical facts of the data are presented in Tables 3-4 and were extrapolated into inferring the individual and combined diagnostic utility of HBME-1, galectin-3, and *BRAF* V600E expression in morphologic differentiation of thyroid neoplasms.

**Table 2:** HBME-1 and galectin-3 expression in PTC, PTH, BRAF positive PTC, all follicular neoplasms, and BRAF positive follicular neoplasms. ** Highly significant as P-value < 0.01, (1% level of significance), * Significant as P-value < 0.05, (5 % level of significance), NS: Non-significant as P-value > 0.05 PTC: papillary thyroid carcinoma; PTH: papillary thyroid hyperplasia; HBME-1: human bone marrow endothelial marker-1

	HBME-1		Galectin-3		
	N	% (P-value)	N	% (P-value)	
Papillary carcinoma (N=33)					
Positive	28	85% (0.000^**^)	25	75.75% (0.000^**^)	
Negative	5	15%	8	24.25%	
Adjacent thyroid tissue with reactive papillary thyroid hyperplasia					
Positive	0	0	0	0	
Negative	33	100% (0.000^**^)	33	100% (0.000^**^)	
BRAF positive papillary thyroid carcinoma (N=9)					
Positive	9	100% (0.000^**^)	9		100% (0.000^**^)
Negative	0	0%	0		0
Follicular carcinoma (N=9)					
Positive	4	44% (0.000^**^)	6	67% (0.000^**^)	
Negative	5	56%	3	33%	
Follicular adenoma (N=20)					
Positive	7	35% (0.000^**^)	5	25% (0.000^**^)	
Negative	13	65%	15	75%	
BRAF positive Follicular neoplasms (N=5)					
Positive	1	20% (0.000^**^)	1	20% (0.000^**^)	
Negative	4	80%	4	80%	

**Table 3:** Individual statistical validation of HBME-1, galectin-3 expression, and BRAF V600E mutation in differentiating among papillary thyroid carcinoma, papillary thyroid hyperplasia, follicular adenoma, and follicular carcinoma. HBME-1: human bone marrow endothelial marker-1; PTC: papillary thyroid carcinoma; PTH: papillary thyroid hyperplasia

Statistic	HBME 1	Galectin 3
Papillary carcinoma		
P-value	p<0.001	p<0.001
Odds ratio	10.27	5.73
Likelihood	28.07	28.07
Sensitivity	84.8%	75.8%
Specificity	63.6%	63.6%
Positive predictive value	67.6%	67.6%
Negative predictive value	80.8	80.8%
Reactive PTH		
P-value	p<0.05	p<0.05
Odds ratio	0.04	0.04
Likelihood	8.962	8.962
Sensitivity	0.0%	0.0%
Specificity	0.0%	0.0%
Positive predictive value	0.0%	0.0%
Negative predictive value	0.0%	0.0%
BRAF positive PTC		
P-value	p<0.001	p<0.05
Odds ratio	15.98	13
Likelihood	20.42	20.42
Sensitivity	98.7%	98.8%
Specificity	0.0%	0.0%
Positive predictive value	75.8%	83.8%
Negative predictive value	0.0%	0.0%
Follicular carcinoma		
P-value	p<0.05	p<0.05
Odds ratio	0.4889	1.74
Likelihood	12.36	12.36
Sensitivity	44.4%	66.7%
Specificity	36.8%	45.6%
Positive predictive value	10%	16.2%
Negative predictive value	80.8%	89.7%
Follicular adenoma		
P-value	p<0.001	p<0.001
Odds ratio	0.23	0.16
Likelihood	25.9	25.9
Sensitivity	35.0%	75.0%
Specificity	28.3%	51.1%
Positive predictive value	17.5%	40.5%
Negative predictive value	50.0%	82.1%
BRAF positive follicular neoplasm		
P-value	p>0.05	p>0.05
Odds ratio	0.15	0.18
Likelihood	5.00	5.00
Sensitivity	80%	20.0%
Specificity	59.0%	41.0%
Positive predictive value	10.0%	2.7%
Negative predictive value	90.0%	86.2%

**Table 4:** Combined statistical validation of HBME-1, galectin-3, and BRAF V600E co-expression in differentiating among papillary thyroid carcinoma, papillary thyroid hyperplasia, follicular adenoma, and follicular carcinoma. HBME-1: human bone marrow endothelial marker-1; PTC: papillary thyroid carcinoma

BRAF in PTC	
P-value	p<0.001
Odds ratio	2.18
Likelihood	26.66
Sensitivity	64.3%
Specificity	100%
Positive predictive value	100%
Negative predictive value	79.5%
HBME 1+Galectin 3 in PTC	
P-value	p<0.001
Odds ratio	7.18
Likelihood	17.48
Sensitivity	88.9%
Specificity	100%
Positive predictive value	100%
Negative predictive value	62.5%
BRAF+HBME 1+Galectin 3 in PTC	
P-value	p<0.001
Odds ratio	12.37
Likelihood	7.71
Sensitivity	100%
Specificity	16.7%
Positive predictive value	28.6%
Negative predictive value	100%
BRAF in follicular neoplasms	
P-value	p<0.05
Odds ratio	0.46
Likelihood	8.96
Sensitivity	100[DR2] %
Specificity	85.9%
Positive predictive value	6.7%
Negative predictive value	100%
BRAF+HBME 1+Galectin 3 in follicular neoplasms	
P-value	p>0.05
Odds ratio	12.00
Likelihood	1.28
Sensitivity	100%
Specificity	38.5%
Positive predictive value	5.9%
Negative predictive value	100%

## Discussion

Mutations in thyroid neoplasms

Morphological assessment alone is often adequate for diagnosing thyroid malignancies. Ancillary studies like IHC and mutation analysis are useful when there are unusual patterns to confirm diagnosis and prognosis.

**Figure 5:**
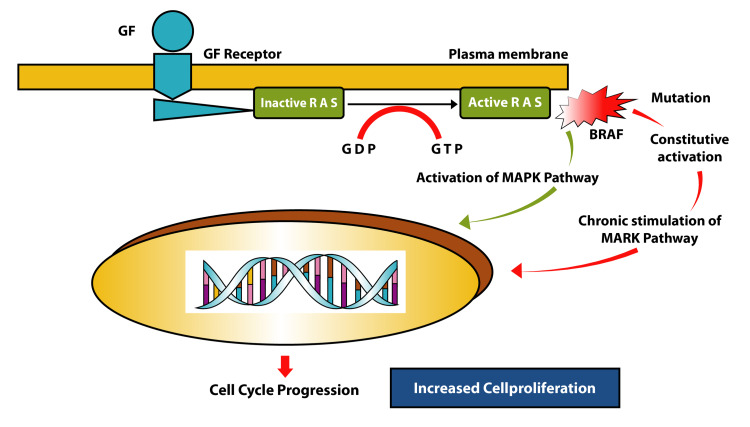
Mechanism of BRAF action. GF: Growth factor; GDP: Guanosine diphosphate; GTP: Guanosine triphosphate; MAPK: Mitogen-activated protein kinase [Original image]

**Figure 6:**
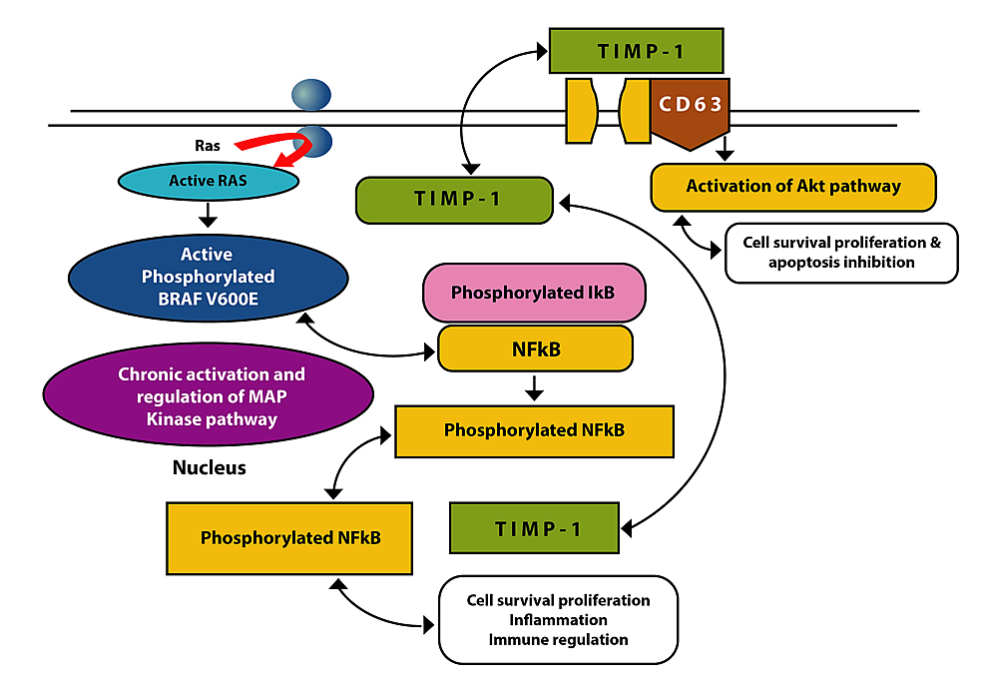
BRAF V600E, NFKb, and TIMP-1 interaction. TIMP: tissue inhibitor of metalloproteinases [Original image]

*BRAF* mutations are very common among thyroid malignancies. The *BRAF* V600E mutation is a transverse mutation resulting in thymine to adenine substitution, which localizes to the kinase domain on exons 11 and 15 of the gene. The *BRAF* V600E gene is situated on chromosome 7 and is very potent in activating the Mitogen-activated protein kinase (MAPK) pathway. The mutation targets the RAS-RAF-MEK-ERK cascade, which is the main pathway of regulation for the growth of cells, proliferation, and differentiation. The mutation activates the RAS family of GTPases, which in turn activates the RAF kinase family complex. This leads to hyperactivation of the BRAF kinase with consequent enhanced downstream signaling. These further activate other relevant parts of the MEK/ERK cascade signaling to over 150 downstream targets. Following this, there is increased transcription of genes that are responsible for cell survival and turnover (Figures 5-6 ) [2].

Apart from acting on the MAPK pathway, the activated *BRAF* oncogene can act along with nuclear factor (NF)-kb, tissue inhibitor of metalloproteinases (TIMP-1), and its cell surface receptor CD63. All these changes trigger molecular events responsible for tumorigenesis [3]. The *BRAF* positive thyroid neoplasms follow a typical phenotype-genotype correlation. They are more common in papillary carcinoma of the thyroid and its variants. *BRAF* V600E mutated papillary carcinomas have distinct morphologic characteristics. The neoplastic cells are plump and eosinophilic with classical nuclear features of PTC. They are unencapsulated lesions with an infiltrative growth pattern and surrounding desmoplasia. They are more prone to metastasis to lymph nodes, extension to extrathyroidal tissue, persistence of the tumor, and recurrence. Thus data available to date suggest that the presence of *BRAF* mutation in a PTC indicates a poor prognosis [3]. Therefore, screening for *BRAF* mutations has an indispensable value as a diagnostic and prognostication marker.

IHC markers

As molecular diagnosis is time-consuming and expensive, there is always a search for other modalities for diagnosis. IHC markers are such that can be used for diagnosis and prognosis. Two such markers for thyroid expression are HBME-1 and galectin-3.

HBME-1

The anti-HBME-1 antibody was first described by Battifora et al. in 1992. It is a monoclonal antibody and recognizes an unknown antigen present in normal tracheal epithelium, microvilli of mesothelioma cells, and adenocarcinoma of breast, lung, and pancreas [4]. HBME-1 shows greater immunostaining with malignant lesions than benign lesions with a membranous or luminal pattern of staining [5].

Extensive studies on HBME-1 have shown that the sensitivity and specificity of HBME-1 in detecting cases of PTC are 80% and 96%, respectively. The diagnostic accuracy of HBME-1 in classical PTC and follicular variant of papillary thyroid carcinoma (FVPTC) is 86% [6]. In a study done by Papotti et al., HBME-1 was positive in 70% of cases of well-differentiated tumors of undetermined malignant potential [7]. Hence, a detailed analysis should be sought in suspicious adenomas or dominant hyperplastic nodules showing HBME- 1 reactivity. These lesions could be possible putative precursors to PTC [7]. HBME-1 positivity in thyroid lesions indicates malignancy. But it does not necessarily indicate papillary differentiation of the lesion [5].

Galectin-3

**Figure 7:**
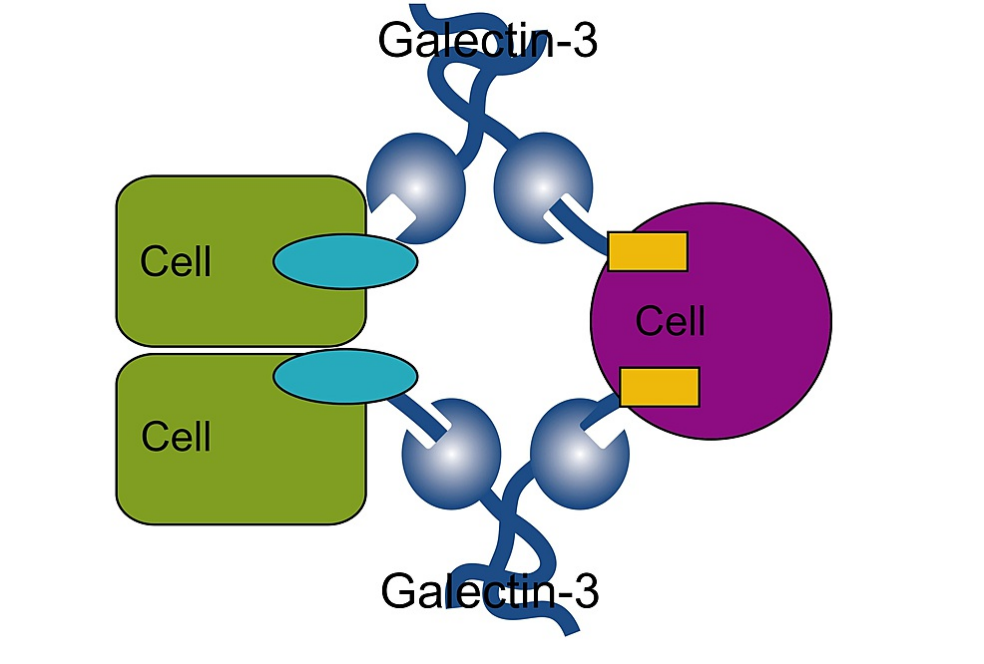
Structure and role of galectin-3 in cell to cell interaction. [Original image]

Galectin-3 is a member of the lectin family and is a beta-galactosidase-binding polypeptide. It is a 31-kDa protein that is constitutively expressed by several epithelial cells and immune cells [8-10]. It plays a role in the differentiation of cells, cell growth, apoptosis, cell adhesion, and interaction with cell-matrix (Figure 7).

Individual and combined utility of HBME-1 and galectin-3 in differentiating PTC from reactive PTH

**Figure 8:**
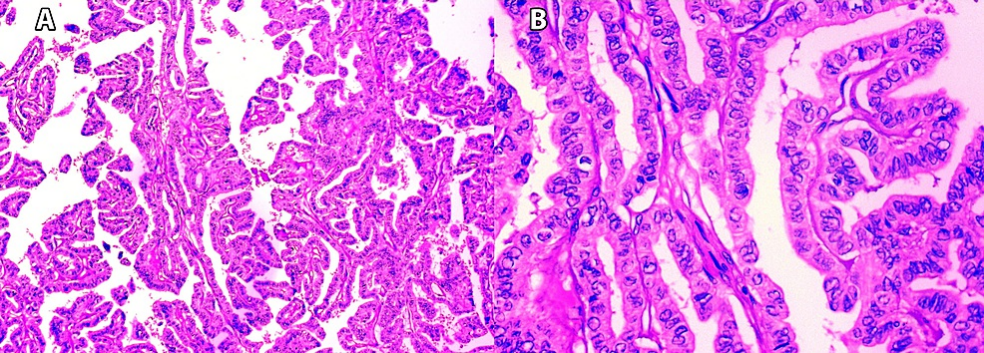
(A) Papillary thyroid carcinoma: the cells show characteristic nuclear findings such as nuclear grooving, nuclear crowding, nuclear overlapping, ground glass chromatin, and intranuclear cytoplasmic pseudoinclusions (H&Ex100); (B) Prominent papillary architecture of the neoplasm with characteristic nuclear features (H&E, x400).

Neoplasia with papillary architecture is often observed in benign and malignant thyroid diseases. Thus, distinguishing between reactive and neoplastic papillary formations may sometimes be difficult [9-11]. Clear morphological characteristics, such as papillary architecture with typically complex branching and characteristic nuclear features, are used in the diagnosis of PTC (Figure 8).

However, distinguishing PTC from PTH and solitary nodules with papillary-like nuclear change is challenging due to tumor heterogeneity. Thus, IHC and ancillary diagnostic techniques are becoming essential for diagnosis [9-11].

HBME-1 was widely reported to be expressed in thyroid tumors, especially in PTC [12,13]. In our study, we found an 85% HBME-1 expression in PTCs and was consistently negative in reactive papillary hyperplasia (Table 2). HBME-1 expression is a very sensitive indicator of PTC (sensitivity = 84.8%), with an odds ratio of 10.27 that also suggests a strong association (Table 3). Our results correlate with those of previous studies wherein 90% of PTCs were immunoreactive for HBME-1 [14]. Rossi et al. reported that HBME-1 alone and combined with galectin-3 and CK19 can help to make the correct distinction between malignant and benign thyroid neoplasms with high diagnostic accuracy [15]. Similarly, Prasad et al. and Rorive et al. have identified HBME-1 as the most specific marker for differentiating carcinomas from benign nodules [16-18]. Our findings are compatible with the literature, and we can define that papillary carcinoma exhibits more intense and diffuse positive staining, which could help rule out reactive papillary hyperplasia with equivocal PTC-like nuclear features.

In our study, galectin-3 showed a strong positive expression in 75.75% (25 out of 33 cases) of PTCs and was consistently negative in reactive papillary hyperplasia (Table 2). Galectin-3 was also strongly associated with PTC (odds ratio = 5.73) with a sensitivity of 75.8% (Table 3). In previous studies, galectin‑3 expression rate was reported to be 64.86%-100.00% in PTC, 45.7%-98.0% in papillary thyroid microcarcinoma (PTMC), 0.0%-52.6% in NG, and 19.0%-38.9% in Hashimoto's thyroiditis (HT) [19-25]. In the present study, 75.75% of the PTC cases were positive for galectin-3 (Table 2). Prasad et al. examined the utility of some markers in thyroid tumors and suggested that galectin-3 was the most sensitive and most accurate marker for carcinomas [16]. Similarly, Saggiorato et al. found that galectin-3 was usually positive in malignant, but negative in benign, thyroid lesions [19]. Contrarily, Park et al. observed that follicular carcinomas stained weakly for galectin-3 [20,26]. Some studies have also demonstrated galectin-3 expression in nodular hyperplasia using multiple techniques [20-22]. Hence, the use of galectin-3 has specific problems because it is expressed in some non-thyroidal tissues, such as histiocytes, squamous cells, and fibroblasts, during degenerative changes in nodular hyperplasia. Thus, we infer that galectin-3 has a low specificity for PTC and benign thyroid lesions and is thus problematic for diagnosing PTC independently. Nevertheless, a strong expression of galectin‑3 along with HBME-1 can potentially diagnose reactive papillary hyperplasia with equivocal PTC-like nuclear features (p < 0.001) (Table 4). The odds ratio of HBME-1 and galectin-3 co-expression is 7.18, which indicates a strong association compared with using either of them alone (Table 4). Similarly, a combined sensitivity of 88.9% and specificity of 100% make the co-expression an ideal marker for differentiating between PTC and reactive PTH (Table 4). Furthermore, the expressions of all the analyzed markers, i.e., HBME-1, galectin-3, and BRAF V600E, were higher in PTC (p < 0.001) than in PTH (Table 4).

Combined utility of HBME-1, galectin-3, and *BRAF* V600E mutation in differentiating FVPTC, follicular adenoma (FA), and solitary nodules with papillary-like nuclear features

The overall expression of HBME-1 and galectin-3 in FAs were seven out of 20 (35%; p < 0.001) and five out of 20 (25%; p < 0.001) cases, respectively (Table-2). The expression of HBME-1 and galectin-3 among nine cases of follicular carcinoma was four out of nine cases (p<0.05) and six out of nine cases (p<0.05), respectively (Table 2). Keeping in view the fact that two cases of follicular carcinoma (FC) had nuclear features of PTC, the expression of HBME-1 and galectin-3 was revised to two out of seven and four out of seven cases, respectively. Thus, in FC cases, HBME-1 had an incidence rate of 28.5%, whereas galectin-3 had 57%. Therefore, we infer that HBME-1 and galectin-3 have significantly higher expression rates (p < 0.001 and p < 0.05, respectively) in PTCs than in FAs and FCs (Tables 2-3).

Five cases of follicular neoplasms were *BRAF* V600E positive. On reassessing these five cases, two had suspicious papillary-like nuclear features and were negative for HBME-1 and galectin-3. Hence, they were excluded from the study. Consequently, the revised incidence of *BRAF* V600E in 27 cases of follicular neoplasms was three out of 37 (11%; p > 0.05) (Table 3). However, the literature had shown none to less than 1% expression of this mutation in follicular neoplasms [1,22]. Thus, the remaining three cases of *BRAF* V600E-positive follicular neoplasms were put through histomorphological and IHC reassessment. Of these, two cases were negative for HBME-1 and galectin-3 in the reassessment and, hence, were also excluded from the study. That left us with one case of follicular neoplasm that showed HBME-1, galectin-3, and *BRAV* V600E positivity (p > 0.05). On reassessment and deeper sectioning of the one remaining case, well-defined nuclear features of PTC were observed. The lesion was subsequently classified as FVPTC. After excluding these cases, the expression of HBME-1 and galectin-3 in *BRAF* V600E-positive follicular neoplasms was 0%. This shows that HBME-1 and galectin-3 were positive only in *BRAF* V600E-positive PTC and FVPTC cases in our study. HBME-1 and galectin-3 were 100% expressed in all *BRAF* V600E-positive PTC cases. Hence, a strong co-expression of HBME-1 and galectin-3 along with *BRAF* V600E expression can differentiate FAs from FVPTC (p < 0.001), especially if papillary features are focal and difficult [18]. The co-expression of HBME-1, galectin-3, and *BRAF* V600E is 100% indicative of PTC and has an odds ratio of 12, which indicates a strong association (Table 3). The diagnostic odds ratio of HBME-1 and galectin-3 is 7.18 in favor of differentiating PTC from follicular neoplasms (Table 3).

Individual utility and controversies of *BRAF* mutations in diagnosing PTC

Several studies have reported that the *BRAF* T1799A mutation may be involved in thyroid carcinoma and that the most mutated locus for this gene lies in exon 15 T1799A [24,25,27]. To date, *BRAF* gene mutations have been found confined to thyroid tumors with papillary differentiation [18,27]. The *BRAF* mutation rate was previously reported to be 29-83% in PTC, 37.5-77.0% in PTMC, and 0.2-5.7% in benign thyroid disease [26,28-33].

In our study, we had 33 PTC cases in which DNA was extracted for 31 cases. Nine cases were *BRAF* V600E positive, giving an incidence of 27% (p < 0.001) (Table 4). The odds ratio of *BRAF* V600E expression is 2.18, which indicates its positive association with PTC (Table 4). HBME-1 and galectin-3 in *BRAF* V600E-positive cases showed a high positivity with an incidence of 100% in *BRAF* V600E mutations (p < 0.001 and p < 0.05, respectively) (Table 3). The overall HBME-1 expression in PTC was 28/33 cases (p < 0.001), whereas that of galectin-3 was 25 out of 33 cases (p < 0.001) (Table 2). HBME-1 and galectin-3 expression in follicular neoplasm was further classified as FVPTC on reassessment.

A significant number of cases (19 out of 28 cases) were positive for HBME-1 and galectin-3 where *BRAF* V600E mutations were negative (p-value). This is also in agreement with the study of Li et al. who found positive expression ratios for HBME-1 and galectin-3 in PTC of 98.8% and 97.6%, respectively. However, *BRAF* mutations were detected in only 40 of 60 (66.7%) PTC specimens 27.

Significant results were observed between the combined co-expression of HBME-1, galectin-3, and *BRAF* mutations in PTC (odds ratio = 12; p < 0.001) (Table 4).

In addition to these, Frasca et al. evaluated *BRAF* mutations in a series of 323 PTC cases and found only 38.6% BRAF positivity [25]. They further suggested the possible link between *BRAF* mutations and environmental carcinogens, which might also explain the heterogeneous results in our series. Some controversies remain on the relationship between *BRAF* mutations and the clinicopathological features of PTC, as demonstrated in our study and reported in the literature cited here [34-41]. Our study found that *BRAF*-positive PTCs are associated with increasing incidence, female predominance, younger age at presentation, and as an incidental finding in thyroidectomy specimens.

## Conclusions

The combined utility of HBME-1, galectin-3, and *BRAF* V600E mutations can help differentiate FVPTC, FA, and solitary nodules with papillary-like nuclear features.

HBME-1 and galectin-3 are expressed in most cases of PTCs irrespective of mutational status and there is a direct correlation between BRAF V600E mutation and HBME-1, galectin-3 expression. However, a larger study is essential among PTCs exclusively with mutational studies and immunomarkers to prove the utility of HBME-1 and galectin-3 as surrogate markers for the mutation.

Although *BRAF* has fairly good sensitivity and positive predictive value, it lacks specificity and hence has a low negative predictive value. Further, there remain some controversies between *BRAF* gene mutation and the clinicopathological features of PTC. Our study suggests that combined IHC staining of HBME-1 and galectin-3 can further improve the sensitivity and specificity of PTC diagnosis, with co-expression of HBME-1 and galectin-3 proving to be a more potent diagnostic criterion. Hence, it may be postulated that while *BRAF* mutation is a significant event in PTC, which may be useful for early diagnosis of the disease, it should be judiciously used with combined panels of IHC markers.
